# Supplementary material for: The prevalence, prevention, and treatment of cardiovascular diseases in Twelve African Countries (2014–2019): An analysis of the World Health Organisation STEPwise approach to chronic disease risk factor surveillance
Source: PLoS One. 2026 Mar 25;21(3):e0320276. doi: 10.1371/journal.pone.0320276 (PMC13016339; doi:10.1371/journal.pone.0320276)
Supplement: S2 File — (DOCX) [file pone.0320276.s002.docx]

**Sample simulation code**

1. *Simulation of discrete variables [(just a few)] like sex, physical activity, smoking status, harmful alcohol use*
2. *Physical activity*

*#Physical activity level*

*set.seed(123) # Set seed for reproducibility*

*# Proportions for each physical activity level*

*pa_proportions <- c("1) Low Level" = 0.16, "2) Moderate level" = 0.20, "3) High level" = 0.64)*

*# Get the indices of NA values in cvd_africa_2024$pa*

*na_indices_pa <- which(is.na(cvd_africa_2024$pa))*

*# Number of NA values*

*n_na_pa <- length(na_indices_pa)*

*# Sample replacement values based on the given proportions*

*replacement_pa_values <- sample(*

*names(pa_proportions), # Possible values ("Low Level", "Moderate Level", "High Level")*

*size = n_na_pa, # Number of values to sample*

*replace = TRUE, # Sampling with replacement*

*prob = pa_proportions # The given probabilities*

*)*

*# Replace the NA values with the sampled values*

*cvd_africa_2024$pa[na_indices_pa] <- replacement_pa_values*

*# Check the updated column*

*table(cvd_africa_2024$pa)*

1. *Harmful alcohol use*

*cvd_africa_2024$alcharm <- factor(cvd_africa_2024$alcharm)*

*set.seed(123) # # Set seed for reproducibility*

*#*

*#Proportions for each category*

*proportions_alcharm <- c("No" = 0.97, "Yes" = 0.03)*

*#*

*#Get the indices of NA values in cvd_africa_2024$alcharm*

*na_indices_alcharm <- which(is.na(cvd_africa_2024$alcharm))*

*#*

*#Number of NA values*

*n_na_alcharm <- length(na_indices_alcharm)*

*#*

*#Sample replacement values based on the given proportions*

*replacement_values_alcharm <- sample(*

*names(proportions_alcharm),*

*size = n_na_alcharm,*

*replace = TRUE,*

*prob = proportions_alcharm*

*)*

1. *Smoking history*

*set.seed(123) # # Set seed for reproducibility*

*#*

*#Proportions for each education level*

*proportions <- c("Current" = 0.1, "Never" = 0.83, "Previous" = 0.07)*

*#*

*#Get the indices of NA values in cvd_africa_2024$edlev*

*na_indices <- which(is.na(cvd_africa_2024$smokehis))*

*#*

*#Number of NA values*

*n_na <- length(na_indices)*

*#*

*#Sample replacement values based on the given proportions*

*replacement_values <- sample(*

*names(proportions), #Possible values ("None", "Primary", etc.)*

*size = n_na, #Number of values to sample*

*replace = TRUE, #Sampling with replacement*

*prob = proportions #The given probabilities*

*)*

*#*

*#Replace the NA values with the sampled values*

*cvd_africa_2024$smokehis[na_indices] <- replacement_values*

*#*

*#Check the updated column*

*table(cvd_africa_2024$smokehis)*

*cvd_africa_2024$smokehis <- factor(cvd_africa_2024$smokehis)*

*var_label(cvd_africa_2024$smokehis) <- "Smoking history"*

1. *Sex*

*set.seed(123) # # Set seed for reproducibility*

*#*

*#Proportions for gender*

*gender_proportions <- c("Female" = 0.49, "Male" = 0.51)*

*#*

*#Get the indices of NA values in cvd_africa_2024$gender*

*na_gender_indices <- which(is.na(cvd_africa_2024$gender))*

*#*

*#Number of NA values*

*n_na_gender <- length(na_gender_indices)*

*#*

*#Sample replacement values based on the given proportions*

*gender_replacements <- sample(*

*names(gender_proportions), #Possible values ("Female", "Male")*

*size = n_na_gender, #Number of values to sample*

*replace = TRUE, #Sampling with replacement*

*prob = gender_proportions #The given probabilities*

*)*

1. *Simulation of continuous variables like age, BMI and cholesterol levels*
2. *BMI*

*set.seed(123)*

*cvd_africa_2024$bmi[is.na(cvd_africa_2024$bmi)] <- rnorm(*

*sum(is.na(cvd_africa_2024$bmi)), #Number of NA values*

*mean = 24.47, # Mean value*

*sd = 11.88 # Standard deviation*

*)*

1. *Cholesterol levels*

*## Set seed for reproducibility*

*set.seed(123)*

*# Define the mean and# Standard deviation*

*mean_cholesterol <- 2.827*

*sd_cholesterol <- 1.502*

*# Find the indices of NA values in cvd_africa_2024$cholesterol*

*na_indices_cholesterol <- which(is.na(cvd_africa_2024$cholesterol))*

*#*

*#Number of NA values*

*n_na_cholesterol <- length(na_indices_cholesterol)*

*#Generate random values from the normal distribution*

*replacement_cholesterol_values <- rnorm(n_na_cholesterol, mean = mean_cholesterol, sd = sd_cholesterol)*

*#Replace the NA values with the generated values*

*cvd_africa_2024$cholesterol[na_indices_cholesterol] <- replacement_cholesterol_values*

*# Replace cholesterol values with their absolute values*

*cvd_africa_2024$cholesterol <- abs(cvd_africa_2024$cholesterol)*

*# Check the updated cholesterol column*

*summary(cvd_africa_2024$cholesterol)*

1. *Age*

*set.seed(123)*

*cvd_africa_2024$age[is.na(cvd_africa_2024$age)] <- rnorm(*

*sum(is.na(cvd_africa_2024$age)), #Number of NA values*

*mean = 37.93, # Mean value*

*sd = 13.52 # Standard deviation*

*)*
